# Enhanced Myogenic Constriction in the SHR Preglomerular Vessels Is Mediated by Thromboxane A2 Synthesis

**DOI:** 10.3389/fphys.2020.00853

**Published:** 2020-07-22

**Authors:** Samera Nademi, Chao Lu, Jeffrey G. Dickhout

**Affiliations:** ^1^Department of Medicine, Division of Nephrology, McMaster University, Hamilton, ON, Canada; ^2^St. Joseph’s Healthcare Hamilton, Hamilton, ON, Canada

**Keywords:** myogenic constriction, renal autoregulation, preglomerular arteries, hypertension, prostaglandin, thromboxane A2, nitric oxide, endothelium

## Abstract

**Background:**

Spontaneously Hypertensive Rats (SHR) have chronically elevated blood pressures at 30 weeks of age (systolic: 191.0 ± 1.0, diastolic: 128.8 ± 0.9). However, despite this chronic malignant hypertension, SHR kidneys remain relatively free of pathology due to having an augmented myogenic constriction (MC). We hypothesized that the enhanced MC in the SHR preglomerular vessels was due to increased prostaglandin and decreased nitric oxide (NO) synthesis, providing renal protection.

**Methods:**

SHR and Wistar Kyoto (WKY) arcuate and mesenteric arteries were treated with indomethacin (prostaglandin synthesis inhibitor), N omega-nitro-L-arginine (L-NNA, NO synthase inhibitor), and nifedipine (L-type calcium channel blocker); and MC was measured in these vessels. The role of endothelium in MC was examined by removing endothelium from WKY and SHR preglomerular and mesenteric arteries using human hair, and measuring MC. We also studied the source of prostaglandin in the SHR by treating endothelium-removed arcuate arteries with indomethacin and furegrelate (thromboxane synthase inhibitor).

**Results:**

MC was enhanced in the SHR preglomerular vessels but not the mesenteric arteries. Indomethacin and LNNA removed the enhanced MC in the SHR. Nifedipine also inhibited MC in both WKY and SHR arcuate and mesenteric arteries. Removing endothelium did not change MC in either arcuate or mesenteric arteries of WKY and SHR rats; and did not remove the augmented MC in the SHR arcuate arteries. Indomethacin and furegrelate decreased MC in endothelium-removed SHR arcuate arteries and obliterated the enhanced MC in the SHR.

**Conclusion:**

The enhanced MC in the SHR arcuate arteries was due to thromboxane A2 synthesis from the tunica media and/or adventitia layers. MC was not dependent on endothelium, but was dependent on L-type calcium channels. Nevertheless, SHR arcuate arteries displayed differential intracellular calcium signaling compared to the WKYs.

## Introduction

In 1901, Bayliss observed that small resistant arteries from different vascular beds of rabbits, cats, and dogs decreased in diameter when he increased intraluminal pressure, and increased diameter with decreasing pressure. Bayliss believed that this response was “myogenic in nature,” and thus later this phenomenon was termed myogenic constriction (MC) (Bayliss, 1902; Saboliæ et al., 1995). MC is stretch-induced reduction in small arteriole diameters in order to regulate the amount of intraluminal blood flow (Kaplan and Palmer, 2001). In the kidneys, renal blood flow autoregulation is an important homeostatic mechanism that protects the delicate glomerular capillaries from fluctuations in the systematic blood pressure, allowing the kidneys to maintain a constant blood flow and glomerular filtration rate (GFR). MC is one of the two mechanisms through which kidneys autoregulate their blood flow (the other mechanism is tubuloglomerular feedback). The significance of MC can be realized from consequences of its’ dysfunction or augmentation. Impaired MC has been observed in variety of diseases such as diabetes, low-renin and salt-sensitive hypertensions (i.e., in African Americans), and chronic kidney disease (CKD) (Carlström et al., 2015; Atala, 2016; Le Goff et al., 2016). On the Contrary, enhanced myogenic constriction protects the kidneys from renal injury. It has been observed that some chronically hypertensive human (i.e., essential hypertensives) and rats (i.e., Spontaneously hypertensive rats, SHR) do not develop significant renal injuries despite highly elevated blood pressures. It appears that this renal protection is due to an augmented MC response in the pre-glomerular vessels (Arendshorst and Beierwaltes, 1979; Imig et al., 1996; Griffin et al., 2001; Loutzenhiser et al., 2002, 2004, 2006; Bidani and Griffin, 2004). We hypothesized that the enhanced MC found in the SHR rats is due to increased prostaglandin H2 (PGH2) and decreased nitric oxide (NO) synthesis in the endothelium compared to their normotensive controls, Wistar Kyoto (WKY) rats. To test this hypothesis, we treated SHR and WKY arcuate and mesenteric arteries with indomethacin and N omega-nitro-L-arginine (LNNA, NO synthase inhibitor) and measured MC. We also examined the role of endothelium in the SHR enhanced MC by removing the endothelium, based on a method that was described by Osol involving the use of human hair (Osol et al., 1989), and measured MC in SHR and WKY arcuate and mesenteric arteries. We utilized both younger and older animals since the augmentation of MC in the SHR may be the result of endothelial dysfunction. We hypnotized this dysfunction would be more severe in the old animals, since they would have been exposed to elevated blood pressure for a longer period of time.

## Materials and Methods

### Animal Studies

Older (30 to 40 weeks old) and younger (12 to 16 weeks old) male WKY and SHR rats were utilized for the vessel studies. Rats’ blood pressure was measured using tail cuff plethysmography (Kent Scientific, CODA system) and their body weights were recorded (Table 1). All rats were bred at McMaster University Central Animal Facility and maintained at St Joseph’s Healthcare Hamilton Animal Facility. All animal work was performed with McMaster University Animal Research Ethics Board’s approval and in accordance with their guidelines. Animals were housed with a 12-hour light-dark cycle and had free access to food and drinking water. After anesthetizing the rodents with isoflurane and perfusing the vasculature with Hank’s basic salt solution (HBSS) to remove blood, arcuate and second branch mesenteric arteries were dissected out of their kidneys and mesenteries, respectively, to conduct vascular studies, as previously (Dickhout and Lee, 1997).

**TABLE 1:** Average age and characteristics of the rats that were used in this study.

	**Older Rats (30–40 weeks)**				**Younger Rats (12–16 weeks)**			
	**Avg. Age**	**SBP**	**DBP**	**BW**	**Avg. Age**	**SBP**	**DBP**	**BW**
	**(weeks)**	**(mmHg)**	**(mmHg)**	**(g)**	**(weeks)**	**(mmHg)**	**(mmHg)**	**(g)**
WKY	34.6 ± 0.5 (*n* = 18)	143.3 ± 6.1 (*n* = 18)	93.4 ± 4.8 (*n* = 18)	439.2 ± 5.6 (*n* = 15)	13.8 ± 0.6 (*n* = 11)	142.7 ± 4.9 (*n* = 5)	85.5 ± 5.8 (*n* = 5)	240.2 ± 30.54 (*n* = 5)
SHR	35.95 ± 0.2 (*n* = 12)	197.4 ± 6.0 (*n* = 12) *	149.1 ± 6.0 (*n* = 12) *	425.2 ± 10.2 (*n* = 12)	13.1 ± 0.4 (*n* = 18)	197.4 ± 7.1 (*n* = 5)*	142.8 ± 8.0 (*n* = 5)*	262.4 ± 39.01 (*n* = 5)

Values are expressed as mean ± SEM; “*n*”: number of animals. Independent two-tailed *T*-test was used to assess significance between WKY and SHR of the same age and parameter. *P ≤ 0.05 was deemed significant.

### Myogenic Response Measurements in Endothelium-Intact Arteries

Renal arcuate and second-branch mesenteric arteries were dissected out of their tissues and transferred into a pressure myograph chamber (PMC) containing 37°C oxygenated HBSS. The PMC was connected to a PS-200 system and a peristaltic pump with a servo-controller to pressurize the arteries (Living Systems, Burlington, VT, United States), and a Leica WILD M3C microscope and Hitachi KP-113 CCD camera to video-record the vessels. In the PMC, the arteries were mounted on a glass micropipette, a blind-sac was created, and the vessels were allowed 30 min to equilibrate to 80 mmHg pressure (P80 mmHg) as previously described (Dickhout and Lee, 1997). To test the functional presence of the endothelium, 3 μM phenylephrine (endothelium-independent vasoconstrictor) was added to pre-constrict the arteries followed by 10 μM carbachol (endothelium-dependent vasodilator), and the diameter-changes were recorded. The chamber was subsequently washed with HBSS and vessels were re-allowed 30 min to equilibrate at P80 mmHg. MC was measured by increasing intraluminal pressure in 20 mmHg increments until 180 mmHg with 5-minute-intervals between each pressure change and the lumen diameters were recorded (E-intact MC). To investigate the effects of prostanoid and NO synthesis, 10 μM indomethacin and 100 μM LNNA, respectively, and independently were added to the PMC, the vessels were allowed 30 min to equilibrate, and MC was measured as described. Passive diameter at each pressure point was measured by replacing HBSS with Ca^2+^-Free HBSS containing 5 mM Ca^2+^ chelator, ethylene glycol tetraacetic acid (EGTA), and re-measuring MC as aforementioned. MC measurements in this study were conducted serially in the following order: normal HBSS (control), drug-treated HBSS, and Ca^2+^-Free HBSS (passive diameter). To investigate the dependency of MC on endothelium, the tunica intima was denuded from the arteries using a human hair.

### Removing Endothelium Using Human Hair

A human hair was glued to a small petri dish and the arteries were moved through the length of the hair about 10 times, according to a method that was described by Osol et al. (1989). The arteries were then re-mounted in the PMC, remnant endothelium was flushed out for 5 min with HBSS, the blind-sac was re-created, and the vessels were allowed 30 min to equilibrate at P80 mmHg. To test the functional absence of the endothelium, 3 μM phenylephrine was added to pre-constrict the arteries followed by 10 μM carbachol and the diameter-changes were recorded. MC was then re-measured (E-removed MC) as described above.

### Myogenic Response Measurements in Endothelium-Removed Arteries

To investigate the effects of prostaglandin and thomboxane A2 (TXA2) synthesis on endothelium-removed vessels, 10 μM indomethacin and 100 μM furegrelate, respectively, and independently were added to the PMC, the vessels were allowed 30 min to adjust, and MC was measured as previously explained. Passive diameter at each pressure point was measured at the end of each experiment by replacing HBSS with Ca^2+^-free HBSS (5 mM EGTA). Physical absence of the endothelium was further investigated.

### Scanning Electron Microscopy (SEM) to Ensure Physical Absence of Endothelium

Endothelium-removed and -intact arcuate arteries were cut in half, fixed with 2% glutaraldehyde (in 0.1 M sodium cacodylate buffer), post-fixed with 2% osmium tetroxide, dehydrated with increasing ethanol concentration, critical point dried, mounted onto microscope stubs, and examined under SEM.

### Data and Statistical Analysis

**FIGURE 1:**
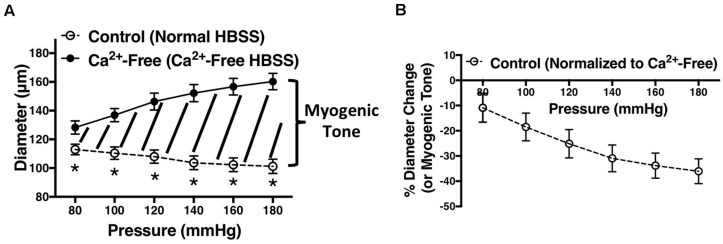
Demonstration of how figures in this manuscript were generated. **(A)** Vessel diameters were recorded at different intraluminal pressures (P80 to P180 mmHg) in the presence of normal HBSS (control) or calcium (Ca^2+^)-free HBSS. The area in between the two graphs (graph of normal HBSS and Ca^2+^-free HBSS) represents myogenic tone. **(B)** Myogenic tone was calculated as percent diameter change by subtracting lumen diameter at each pressure point in Ca^2+^-free HBSS (D_n (Ca2+*free*)_) from the lumen diameter at the same pressure point in normal HBSS (D_n_), divided by the diameter in Ca^2+^-free HBSS (D_n (Ca2+*free*)_), and multiplied by 100 (% Diameter Change $=\left(\frac{D_{n-}\,D_{n\,\left(\mathrm{Ca}-\mathrm{Free}\right)}}{D_{n\,\left(\mathrm{Ca}-\mathrm{Free}\right)}}\right)*100$). Two-way ANOVA and Holm-Sidak *post hoc* test were used to assess significance between two population means at each pressure point; denoted by *P ≤ 0.05. Graphs were made using WKY arcuate arteries. N_*WKY*_ = 5, *n*_*WKY*_ = 5 (“N”: number of vessels, “*n*”: number of animals).

Percent diameter change was calculated by subtracting lumen diameter at each pressure point in Ca^2+^-free HBSS (D_n (Ca2+*free*)_) from the lumen diameter at the same pressure point in normal HBSS (D_n_), divided by the diameter in Ca^2+^-free HBSS (D_n (Ca2+*free*)_), and multiplied by 100 (% Diameter Change $={\left(\frac{D_{n-}\,D_{n\,\left(C\,a-F\,r\,e\,e\right)}}{D_{n\,\left(C\,a-F\,r\,e\,e\right)}}\right)}^{*}\,100$); this is illustrated in Figures 1A,B. Percent relaxation due to carbachol was calculated by subtracting lumen diameter at P80 mmHg after adding carbachol (D_after_) from lumen diameter before adding carbachol (D_before_), divided by diameter before carbachol (D_before_), and multiplied by 100 (% Carbachol Relaxation = ${\left(\frac{D_{b\,e\,f\,o\,r\,e}-D_{a\,f\,t\,e\,r}}{D_{b\,e\,f\,o\,r\,e}}\right)}^{*}\,100$). Paired *t*-test was used to compare carbachol-induced responses before and after endothelium removal. Independent *t*-test was used to compare WKY and SHR population means. Two-way ANOVA was used to compare MC from different rat strains or treatments. If differences were found by ANOVA, *post hoc* comparisons at different pressure points (Holm-Sidak *post hoc* test) were conducted to determine differences in the means. Significant differences were evaluated using GraphPad prism and 95% confidence intervals. *P*-values less than or equal to 0.05 were deemed statistically significant.

### Reagents

The following reagents were purchased from Sigma-Aldrich: phenylephrine (P6126), carbachol (C4382), furegrelate (F3764), indomethacin (I7378), LNNA (N5501), and EGTA (E3889).

## Results

### Effects of Inhibiting PGH2 and NO Synthesis on Endothelium-Intact Vessels Derived From Older Rats

**FIGURE 2:**
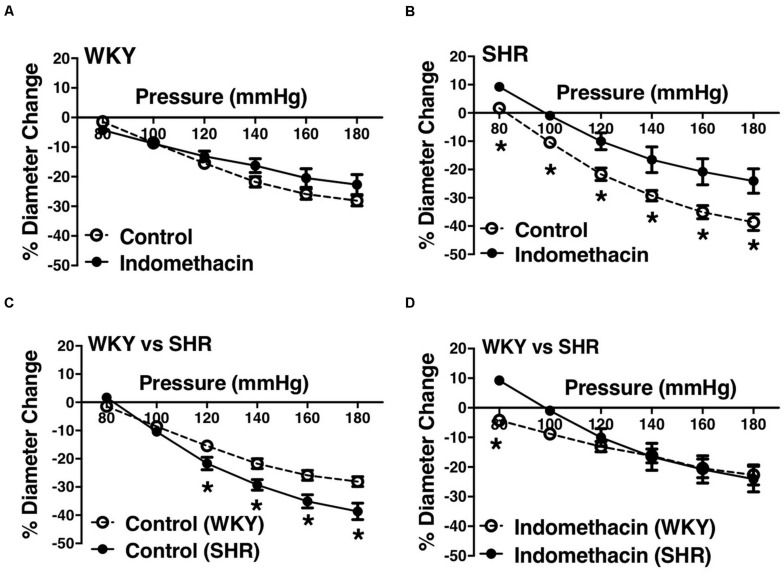
Effects of inhibiting cyclooxygenase 1 and 2 (prostaglandin H2 synthesis) in WKY and SHR preglomerular arteries (30–40 weeks-old rats). **(A)** Effect of indomethacin (10 μM) on WKY arcuate artery myogenic constriction (MC). **(B)** Effect of indomethacin (10 μM) on SHR arcuate artery MC. **(C)** Comparison of MC between WKY and SHR arcuate arteries. **(D)** Comparison of MC between WKY and SHR arcuate arteries that were treated with 10 μM indomethacin. Two-way ANOVA and Holm-Sidak *post hoc* test were used to assess significance; denoted by *P ≤ 0.05. N_*WKY*_ = 9, N_*SHR*_ = 5, *n*_*WKY*_ = 7, *n*_*SHR*_ = 5 (“N”: number of vessels, “*n*”: number of animals).

**FIGURE 3:**
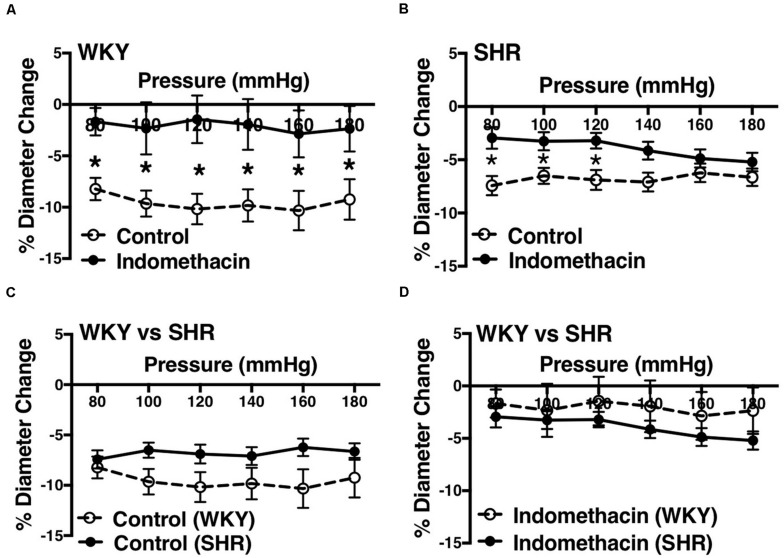
Effects of inhibiting cyclooxygenase 1 and 2 (prostaglandin H2 synthesis) in WKY and SHR mesenteric arteries (30–40 weeks-old rats). **(A)** Effect of indomethacin (10 μM) on WKY mesenteric artery myogenic constriction (MC). **(B)** Effect of indomethacin (10 μM) on SHR mesenteric artery MC. **(C)** Comparison of MC between WKY and SHR mesenteric arteries. **(D)** Comparison of MC between WKY and SHR mesenteric arteries that were treated with 10 μM indomethacin. Two-way ANOVA and Holm-Sidak *post hoc* test were used to assess significance; denoted by *P ≤ 0.05. N_*WKY*_ = 8, N_*SHR*_ = 17, *n*_*WKY*_ = 5, *n*_*SHR*_ = 6 (“N”: number of vessels, “*n*”: number of animals).

**FIGURE 4:**
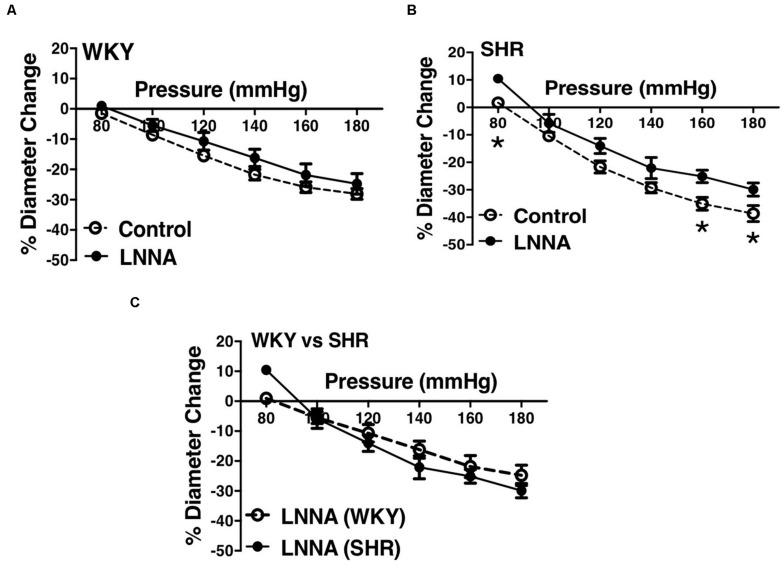
Effects of nitric oxide synthase inhibition on MC of preglomerular arteries in WKY and SHR rats (30–40 weeks-old rats). **(A)** Effect of N omega-nitro-L-arginine (LNNA, 100 μM) on myogenic constriction (MC) of WKY arcuate arteries. **(B)** Effect of LNNA (100 μM) on MC of SHR arcuate arteries. **(C)** Comparing MC in WKY and SHR arcuate arteries that were treated with LNNA (100 μM). Two-way ANOVA and Holm-Sidak *post hoc* test were used to assess significance; denoted by *P ≤ 0.05. N_*WKY*_ = 7, N_*SHR*_ = 6, *n*_*WKY*_ = 7, *n*_*SHR*_ = 6 (“N”: number of vessels, “*n*”: number of animals).

**FIGURE 5:**
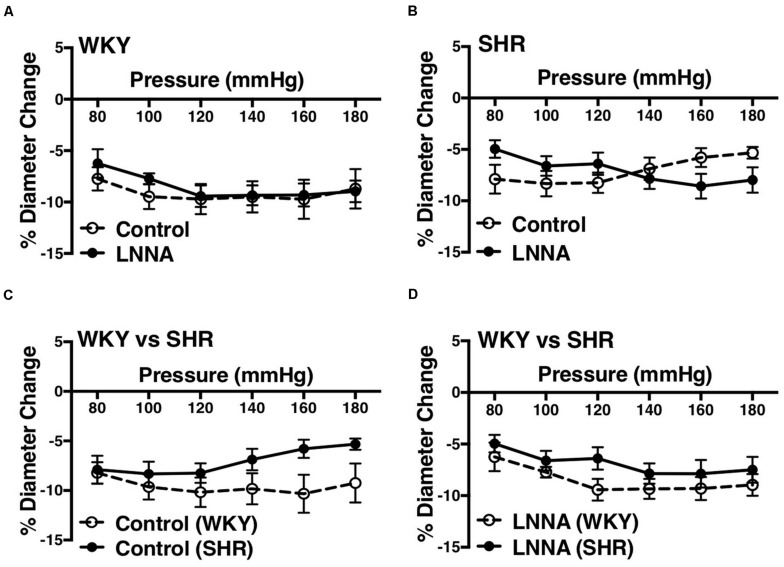
Effects of nitric oxide synthase inhibition on MC of mesenteric arteries in WKY and SHR rats (30–40 weeks-old rats). **(A)** Effect of N omega-nitro-L-arginine (LNNA, 100 μM) on myogenic constriction (MC) of WKY mesenteric arteries. **(B)** Effect of LNNA (100 μM) on MC of SHR mesenteric arteries. **(C)** Comparison of MC between WKY and SHR mesenteric arteries. **(D)** Comparing MC in WKY and SHR mesenteric arteries that were treated with LNNA (100 μM). Two-way ANOVA and Holm-Sidak *post hoc* test were used to assess significance; denoted by *P ≤ 0.05. N_*WKY*_ = 7, N_*SHR*_ = 6, *n*_*WKY*_ = 5, *n*_*SHR*_ = 6 (“N”: number of vessels, “*n*”: number of animals).

We used indomethacin, a non-selective cyclooxygenase (COX-1 and COX-2) inhibitor, to block PGH2 synthesis (Lucas, 2016). Indomethacin reduced MC in the SHR but not the WKY arcuate arteries (Figures 2A,B). SHR arcuate arteries demonstrated an enhanced MC compared to the WKY arteries (Figure 2C), and the augmented MC in the SHR was abolished by indomethacin treatment (Figure 2D). Comparably, indomethacin decreased MC in both SHR and WKY mesenteric arteries (Figures 3A,B). Nevertheless, an enhanced MC was not observed in the SHR mesenteric arteries compared to the WKY (Figure 3C). Also, indomethacin-treated SHR and WKY mesenteric vessels showed similar MC (Figure 3D). We also investigated the effects of blocking nitric oxide synthase (NOS) by LNNA on MC in the SHR and WKY rats. Similar to the indomethacin, LNNA reduced MC in the SHR arcuate arteries at P80, P160, and P180 mmHg; but did not change MC in the WKY pre-glomerular vessels (Figures 4A,B). LNNA also removed the augmented MC in the SHR arcuate arteries compared to the WKY (Figure 4C). In mesenteric arteries, LNNA treatment did not change MC in either WKY or SHR, and there were no differences between WKY and SHR LNNA-treated vessels (Figures 5A–D).

### Effects of Inhibiting L-Type Calcium Channels on MC in Endothelium-Intact Vessels

**FIGURE 6:**
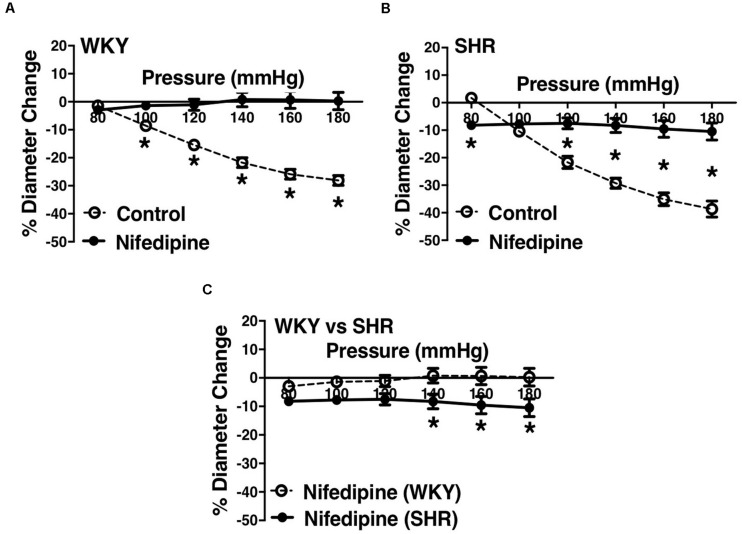
Effects of L-type calcium channel blockade on arcuate arteries of WKY and SHR older rats (30–40 weeks-old). **(A)** Effect of nifedipine (10 μM) on myogenic constriction (MC) of WKY arcuate arteries. **(B)** Effect of nifedipine (10 μM) on MC of SHR arcuate arteries. **(C)** Comparing MC in WKY and SHR preglomerular arteries that were treated with nifedipine (10 μM). Two-way ANOVA and Holm-Sidak *post hoc* test were used to assess significance; denoted by *P ≤ 0.05. N_*WKY*_ = 7, N_*SHR*_ = 5, *n*_*WKY*_ = 4, *n*_*SHR*_ = 4 (“N”: number of vessels, “*n*”: number of animals).

**FIGURE 7:**
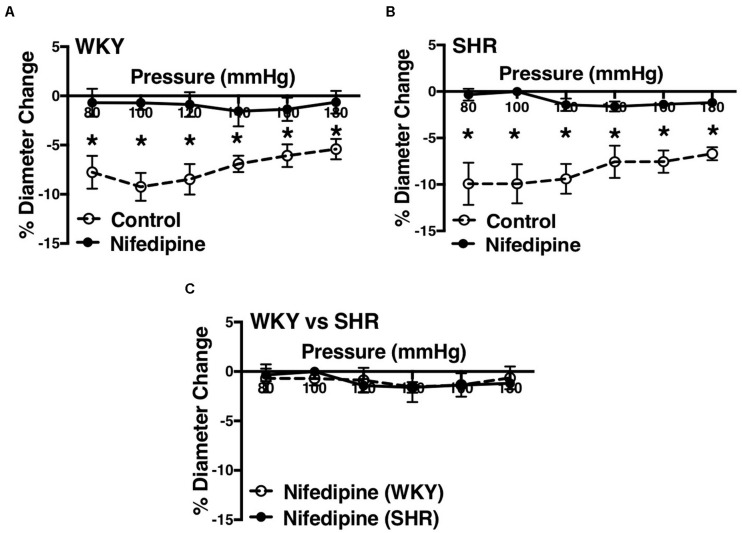
Effects of L-type calcium channel blockade on mesenteric arteries of WKY and SHR older rats (30–40 weeks-old). **(A)** Effect of nifedipine (1 μM) on myogenic constriction (MC) of WKY mesenteric arteries. **(B)** Effect of nifedipine (1 μM) on MC of SHR mesenteric arteries. **(C)** Comparing MC in WKY and SHR mesenteric arteries that were treated with nifedipine (1 μM). Two-way ANOVA and Holm-Sidak *post hoc* test were used to assess significance; denoted by *P ≤ 0.05. N_*WKY*_ = 9, N_*SHR*_ = 8, *n*_*WKY*_ = 5, *n*_*SHR*_ = 4 (“N”: number of vessels, “*n*”: number of animals).

**FIGURE 8:**
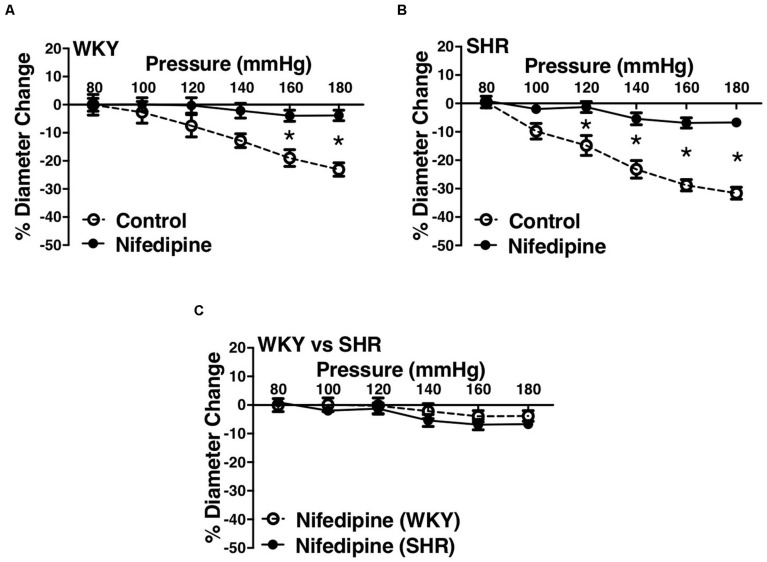
Effects of L-type calcium channel blockade on arcuate arteries of WKY and SHR younger rats (12–16 weeks-old). **(A)** Effect of nifedipine (10 μM) on myogenic constriction (MC) of WKY arcuate arteries. **(B)** Effect of nifedipine (10 μM) on MC of SHR arcuate arteries. **(C)** Comparing MC in WKY and SHR arcuate arteries that were treated with nifedipine (10 μM). Two-way ANOVA and Holm-Sidak *post hoc* test were used to assess significance; denoted by *P ≤ 0.05. N_*WKY*_ = 5, N_*SHR*_ = 5, *n*_*WKY*_ = 5, *n*_*SHR*_ = 5 (“N”: number of vessels, “*n*”: number of animals).

L-type Ca^2+^ channel blocker, nifedipine, significantly reduced MC in both WKY and SHR arcuate (Figures 6A,B) and mesenteric arteries (Figures 7A,B) of older rats. Nonetheless in older rats, nifedipine-treated SHR arcuate arteries showed significantly more MC compared to the WKY arcuate arteries (Figure 6C) at P140 to P180 mmHg; this difference was not observed in the SHR and WKY mesenteric arteries (Figure 7C). Further, we also investigated the effects of nifedipine in arcuate arteries from younger WKY and SHR rats (12–16 weeks old). Similar to the older rats (30–40 weeks old), nifedipine blocked MC in both WKY and SHR arcuate arteries of the younger rats (Figures 8A,B). However, there were no differences between nifedipine-treated WKY and SHR arcuate arteries (Figure 8C).

### Endothelium Was Successfully Removed Using Human Hair

**FIGURE 9:**
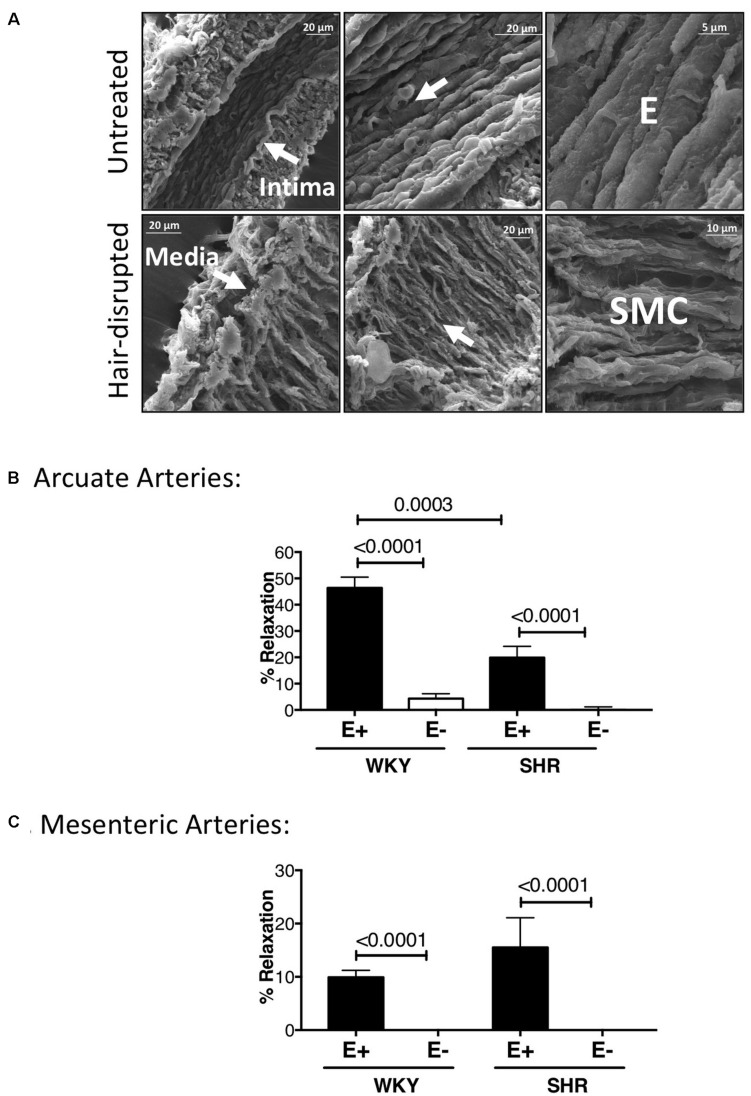
Scanning Electron Micrographs and carbachol treatments demonstrating physical and functional removal of endothelium. **(A)** Scanning Electron Microscopy (SEM) showing intact tunica intima (consisting of endothelium, E) and media (consisting of smooth muscle cells) in untreated, but only tunica media in hair-disrupted WKY arcuate arteries. Micrographs were magnified between 700 to 15000 times. **(B)** Percent carbachol-induced relaxation (10 μM) in WKY and SHR arcuate arteries that were pre-constricted with 3 μM phenylephrine at P80 mmHg. **(C)** Percent carbachol-induced relaxation (10 μM) in WKY and SHR mesenteric arteries that were pre-constricted with 3 μM phenylephrine at P80 mmHg. Paired *t*-test was used to compare endothelium-intact (E+) and endothelium-removed (E-) arteries in the same animal strain. Unpaired *t*-test was used to compare E + and E- arteries in different animal strains. *P ≤ 0.05; for arcuate arteries: N_*WKY*_ = 10, N_*SHR*_ = 10, *n*_*WKY*_ = 5, *n*_*SHR*_ = 4; for mesenteric arteries: N_*WKY*_ = 11, N_*SHR*_ = 5, *n*_*WKY*_ = 5, *n*_*SHR*_ = 4; (“N”: number of vessels; “*n*”: number of animals).

Scanning Electron Microscopy micrographs showed complete physical absence of the endothelium in WKY arcuate arteries. The endothelium was oriented longitudinally, whereas the smooth muscle cell (SMC) layer was oriented cross-sectionally (Figure 9A). WKY and SHR pre-constricted arcuate arteries (by 3 μM phenylephrine) showed almost no relaxation to 10 μM carbachol after removing the endothelium. WKY endothelium-intact arcuate vessels showed significantly more relaxation compared to SHR endothelium-intact vessels (Figure 9B). Pre-constricted mesenteric arteries (by 3 μM phenylephrine) in WKY and SHR also showed lack of relaxation to 10 μM carbachol after removing the endothelium. There were no statistically significant differences between WKY and SHR endothelium-intact carbachol-induced relaxation (Figure 9C).

### Effects of Removing Endothelium on Myogenic Constriction in the WKY and SHR

**FIGURE 10:**
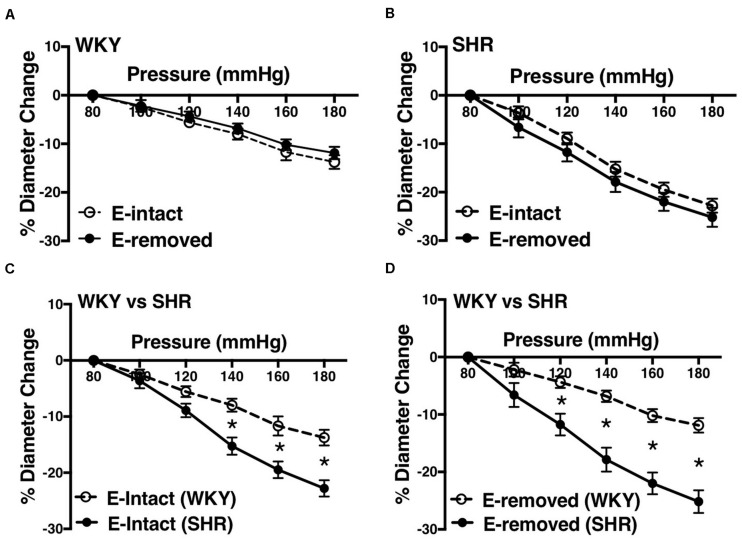
Effects of removing endothelium from WKY and SHR preglomerular arteries using human hair (30–40 weeks-old rats). **(A)** Effect of removing endothelium in WKY arcuate arteries. **(B)** Effect of removing endothelium in SHR arcuate arteries. **(C)** Comparison of myogenic constriction (MC) in endothelium-intact (E-intact) WKY and SHR arcuate arteries. **(D)** Comparison of MC in endothelium-removed (E-removed) WKY and SHR arcuate arteries. Two-way ANOVA and Holm-Sidak *post hoc* test was used to assess significance; denoted by *P ≤ 0.05. N_*WKY*_ = 10, N_*SHR*_ = 10, *n*_*WKY*_ = 5, *n*_*SHR*_ = 4; (“N”: number of vessels; “*n*”: number of animals).

**FIGURE 11:**
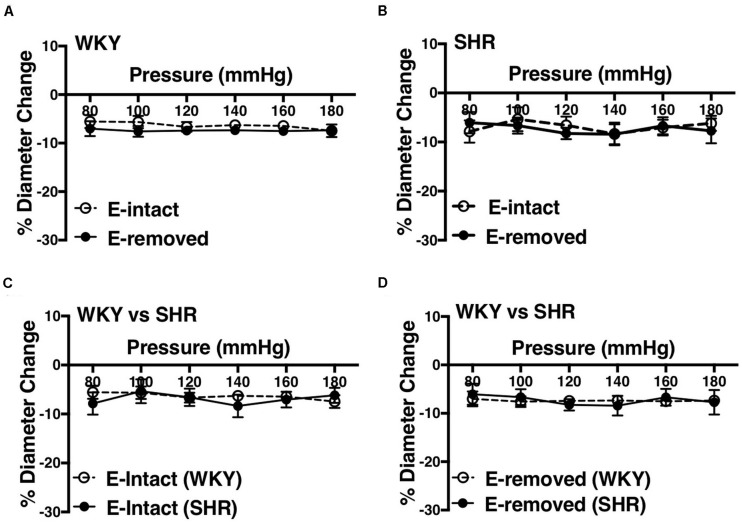
Effects of removing endothelium from WKY and SHR mesenteric arteries using human hair (30–40 weeks-old rats). **(A)** Effect of removing endothelium in WKY mesenteric arteries. **(B)** Effect of removing endothelium in SHR mesenteric arteries. **(C)** Comparison of myogenic constriction (MC) in endothelium-intact (E-intact) WKY and SHR mesenteric arteries. **(D)** Comparison of MC in endothelium-removed (E-removed) WKY and SHR mesenteric arteries. Two-way ANOVA and Holm-Sidak *post hoc* test was used to assess significance; denoted by *P ≤ 0.05. N_*WKY*_ = 11, N_*SHR*_ = 5, *n*_*WKY*_ = 6, *n*_*SHR*_ = 4; (“N”: number of vessels; “*n*”: number of animals).

Removing endothelium did not change MC in the WKY and SHR arcuate (Figures 10A,B) and mesenteric arteries (Figures 11A,B). Moreover, SHR endothelium-intact arcuate arteries demonstrated enhanced MC compared to the WKY endothelium-intact arcuate arteries at P140 to P180 mmHg (Figure 10C), but this enhancement was absent in the SHR mesenteric arteries (Figure 11C). Denuding endothelium did not remove the augmented MC in the SHR arcuate arteries (Figure 10D). Furthermore, MC was similar in endothelium -removed WKY and SHR mesenteric arcades (Figure 11D).

### Effects of Inhibiting Prostaglandin H2 and Thromboxane A2 Synthesis in Endothelium-Removed Arcuate Arteries of Younger SHRs

**FIGURE 12:**
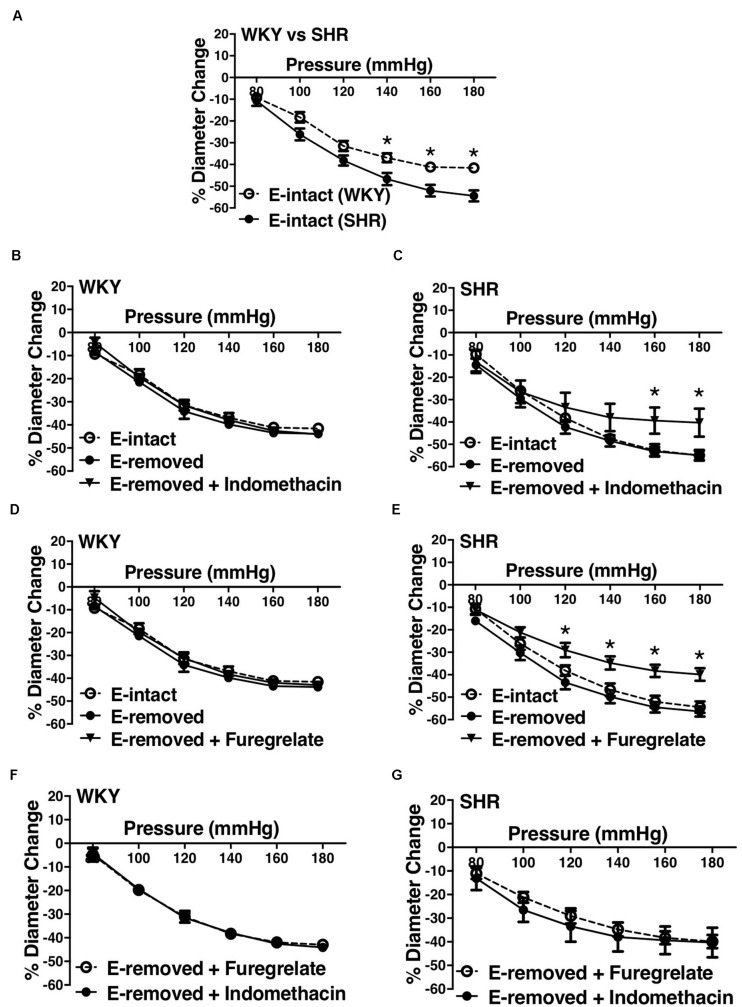
Effects of inhibiting cyclooxygenase 1 and 2 (prostaglandin H2 synthesis) and thromboxane synthetase on arcuate arteries of young WKY and SHR rats (12–16 weeks-old). **(A)** Myogenic constriction (MC) comparison between endothelium-intact (E-intact) WKY and SHR arcuate arteries. **(B,C)** Comparing WKY and SHR arcuate arteries with E-intact, endothelium-removed (E-removed), and E-removed that were treated with indomethacin (prostaglandin H2 synthesis inhibitor, 10 μM). Star,“*”, depicts significant differences between indomethacin-treated vessels with when they had their E-removed and E-intact (*P* ≤ 0.05). **(D,E)** Comparing WKY and SHR arcuate arteries with E-intact, E-removed, and E-removed that were treated with furegrelate (thromboxane A2 synthesis inhibitor, 100 μM). Star,“*”, depicts significant differences between furegrelate-treated vessels with when they had their E-removed and E-intact (*P* ≤ 0.05). **(F,G)** Comparing WKY and SHR E-removed arcuate arteries that were treated with either indomethacin or furegrelate. Two-way ANOVA and Holm-Sidak *post hoc* test was used to assess significance. N_*WKY*_ = 9, N_*SHR*_ = 10, *n*_*WKY*_ = 6, *n*_*SHR*_ = 9; (“N”: number of vessels; “*n*”: number of animals).

**FIGURE 13:**
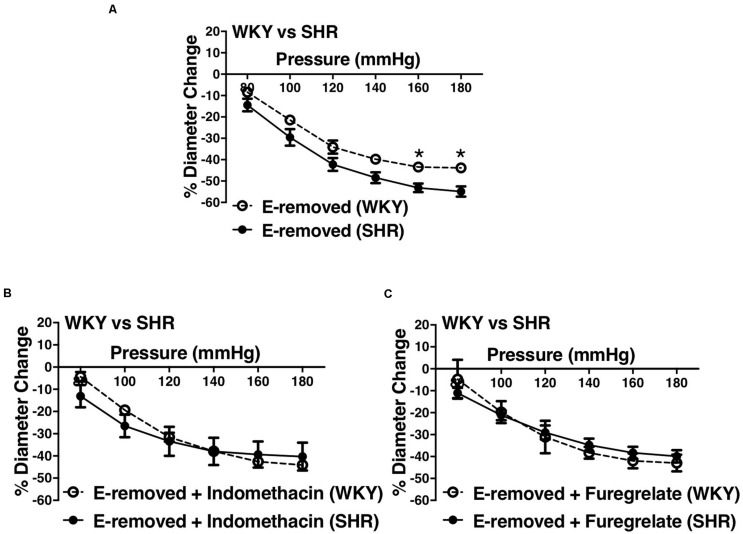
Enhanced myogenic constriction (MC) in the young SHR arcuate arteries, compared to the WKY, was abolished with inhibiting prostaglandin H2 synthesis (by indomethacin) and thromboxane A2 synthesis (by furegrelate) independent from endothelium. **(A)** MC is enhanced in the endothelium-removed (E-removed) SHR arcuate arteries compared with E-removed WKY arcuate arteries at high intraluminal pressures. **(B)** Treating E-removed SHR arcuate arteries with 10 μM indomethacin abolished the enhanced MC compared to the WKY arcuate arteries. **(C)** Treating E-removed SHR arcuate arteries with 100 μM furegrelate abolished the enhanced MC compared to the WKY arcuate arteries. Two-way ANOVA and Holm-Sidak *post hoc* test was used to assess significance; denoted by *P ≤ 0.05. N_*WKY*_ = 9, N_*SHR*_ = 10, *n*_*WKY*_ = 6, *n*_*SHR*_ = 9; (“N”: number of vessels; “*n*”: number of animals).

Similar to the older rats (30–40 weeks old), arcuate arteries of younger SHR rats (12–16 weeks old) showed enhanced MC at P140, P160, and P180 mmHg (Figure 12A). Inhibiting PGH2 synthesis by indomethacin in endothelium-removed arcuate arteries significantly decreased MC at P160 and P180 mmHg in the SHR (compared to both E-intact and E-removed vessels) but did not change MC in the WKY (Figures 12B,C). Similarly, inhibiting TXA2 synthesis by furegrelate in endothelium-removed arcuate arteries also significantly decreased MC at P120 to P180 mmHg (compared to both E-intact and E-removed vessels), but did not change MC in the WKY (Figures 12D,E). There were no differences in endothelium-removed arcuate arteries that had been treated with indomethacin or furegrelate in either WKY or SHR (Figures 12F,G). Similar to the older rats (Figure 10D), MC was still enhanced in the endothelium-removed SHR arcuate arteries compared to the endothelium-removed WKY arcuate arteries (Figure 13A). Treating SHR endothelium-removed preglomerular arteries with indomethacin and furegrelate obliterated the enhanced MC in these vessels compared to the WKY arcuate arteries (Figures 13B,C).

## Discussion

**FIGURE 14:**
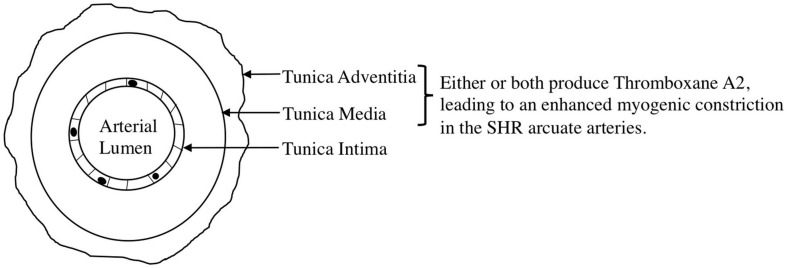
Proposed mechanism for the enhanced myogenic constriction in the SHR arcuate arteries. Tunica intima, Tunica media, and tunica adventitia are composed of endothelial cells, smooth muscle cells, and connective tissue, respectively.

We observed an augmented MC in the SHR pre-glomerular arteries compared to the WKY pre-glomerular arteries of both younger and older rats (Figures 2C, 4C, 10C, 12A). This augmented MC was absent in the SHR mesenteric vessels (Figures 3C, 5C, 11C). We also found that inhibiting prostaglandin synthesis (by indomethacin) obliterated the enhanced MC that was observed in the SHR preglomerular arteries (Figure 2D) by reducing MC in these vessels (Figure 2B). L-type Ca^2+^ channel blocker, nifedipine, reduced MC in all vessels (Figures 6A,B, 7A,B, 8A,B), although SHR arcuate arteries of older rats showed more myogenic tone than WKY arcuate arteries after treating with nifedipine (Figure 6C). Our work showed that MC in WKY and SHR arcuate and mesenteric arteries is independent of endothelium as removing endothelium did not change MC (Figures 10A,B, 11A,B). As well, removing endothelium did not eradicate the enhanced MC in the SHR preglomerular vessels (Figure 10D), suggesting that this augmentation is endothelium-independent. Our results also showed that indomethacin (PGH2 synthesis inhibitor) and furegrelate (TXA2 synthase inhibitor) abolished the enhanced MC in the SHR independently from endothelium (Figures 12C,E) suggesting that the source of TXA2 synthesis in the SHR pre-glomerular arteries is the tunica media and/or adventitia layers, and not the endothelium (Figure 14). In omental arteries, thromboxane synthase has been found in both the tunica media and adventitia layers in both normal and preeclamptic women (Mousa et al., 2012). This is the first study, to our knowledge, to show that MC is independent of endothelium in arcuate and mesenteric arteries of WKY and SHR rats. We were also the first to show that the enhanced MC in the SHR arcuate arteries was due to increased TXA2 synthesis.

### The Enhanced Myogenic Constriction in the SHR

Several studies investigated the reason for the observed enhanced MC during chronic hypertension in different vascular beds. Huang et al. (1993) examined WKY and SHR cremaster muscle arteriole responses to indomethacin (PGH2 synthesis inhibitor) and SQ 29,548 (PGH2 receptor blocker), as well as the effects of endothelium removal (Huang et al., 1993). Contrary to our results, they suggested that increased PGH2 production from endothelium was responsible for the observed enhanced MC in the SHR cremaster muscle arterioles. This could be because different vascular beds display different vasoactive mechanisms in the endothelium and vascular SMCs (Ferrer et al., 1995; Thorin et al., 1997; Hill et al., 2001). Other studies conducted in skeletal muscle arterioles and aorta showed that in hypertension, endothelium synthesizes endothelin (Schiffrin and Thibault, 1991; Lemne et al., 1994; Schiffrin et al., 1997) and TXA2 (formed from PGH2) (Mayhan, 1992; Ungvari and Koller, 2000) which increase sensitivity of smooth muscle contractile apparatus, inducing greater contractions in response to similar raise in intracellular calcium in the SHR SMC layer compared to the Wistar Rats (WR) (Ungvari and Koller, 2000). This is consistent with our findings that also suggest a heightened intracellular calcium response in the SHR preglomerular arteries versus the WKY, as nifedipine-treated SHR vessels showed higher myogenic tone compared to the WKY nifedipine-treated vessels (Figure 6C). This enhanced sensitivity of SHR pre-glomerular arteries to intracellular calcium-rise may be due to activation of Rho-kinase pathway in SMCs (Uehata et al., 1997; Homma et al., 2013). Moreover, diminished NO signaling in skeletal muscle arterioles of hypertensive rats (i.e., Zucker Diabetic Fatty rats) has been shown to contribute to the exaggerated MC (Lesniewski et al., 2008), which is contrary to our finding that NO synthesis may have a small constrictor effect on the SHR arcuate arteries at high intraluminal pressures (P160 and P180 mmHg) (Figure 4B). No is predominantly produced by the endothelium and is very unstable with a half-life of seconds (Butler et al., 1995; Schini, 1996; Jeremy et al., 1999). After being produced, NO can rapidly react with oxygen species (O_2_, O_2_^–^ and H_2_O_2_) and thiol groups to produce nitrite, nitrate, peroxynitrite (ONOO), and nitrosothiols. The effects of NO on vessels can be mediated by the potential presence of thiol and oxygen species (Darley-Usmar et al., 1995; Jeremy et al., 1999). The small vasoconstrictor effects of NO (at high intraluminal pressures) that was observed in this study may be due to the reaction of NO with superoxide to create peroxynitrite in the SHR vessels (Carlisle et al., 2016) and thereby augmenting constrictor responses (Bagi et al., 2002). The resulting peroxynitrite may promote the release of TXA2 leading to arteriolar constriction, as was shown in gracilis muscle arterioles of hyperhomocysteinemia (induced by methionine diet) mouse model (Bagi et al., 2002). As well, in rat models of high-fat high-sucrose (HFHS) and levothyroxine (L-T_4_) diets, endothelium-denuded thoracic aortic rings from HFHS + LT_4_ rats showed stronger vasoconstriction than HFHS rats due to increase NO and superoxide production that led to peroxynitrite formation, independent from the endothelium (Hu et al., 2014). Other contributing mechanisms to the enhanced MC have also been reported such as increased superoxide generation through activation of Nicotinamide Adenine Dinucleotide Phosphate (NADPH) oxidase in afferent arterioles (Ren et al., 2010); increased activation of angiotensin II type 1 receptor in cremaster muscle arteries (Hong et al., 2017); and increased production of 20-hydroxyeicosatrienoic acid (20-HETE) through activation of cytochrome P450 in afferent and cerebral arterioles (Imig et al., 1993, 1994, 1996; Szarka et al., 2017). Consistent with our observation of an augmented MC in the SHR arcuate arteries, (Imig et al., 1993), found an enhanced MC in the SHR (4-week-old prehypertensive SHRs) interlobular as well as proximal and distal portions of the afferent arterioles of juxtamedullary glomeruli. The researchers also discovered that inhibiting cytochrome P-450 enzyme (with ketoconazole or 7-ethoxyresorufin) had variable effects in different segments of the preglomerular arteries. In interlobular, proximal afferent, and distal afferent arterioles, inhibiting cytochrome P-450 did not remove, removed, and partially removed the enhanced MC in the SHR arterioles, respectively, compared to the WKY arteries (Imig et al., 1993). These findings suggest that cytochrome P-450 metabolites of arachidonic acid have a critical role in the enhanced MC in the SHR preglomerular vessels (Imig et al., 1996). To investigate if the enhanced MC in the SHR vessels is endothelium-dependent, we removed the endothelium using human hair.

### Removing the Endothelium

Endothelium can be removed from blood vessels by chemical or physical means. Chemical methods include using collagenase, elastase, or antibodies. Collagenase and elastase are enzymes that dissolve intercellular matrix between endothelium, but might also damage the vascular SMC layer (Osol et al., 1989). Antibodies against specific endothelial antigens may also not completely remove the endothelium (Juncos et al., 1995). Physical methods of denuding endothelium involve abrading the inner surface of the vessel by an applicator such as cotton, filter paper (Molina et al., 1992), wood, wire, air bolus, or human hair; most of which are fragile or difficult to apply to small arteries (Osol et al., 1989) such as preglomerular vessels. Air bolus injections also are appropriate methods for large arteries, but may not completely remove the endothelium in small arterioles (Ralevic et al., 1989). In 1989, Osol et al. suggested an effective method for removing endothelium from small arteries involving human hair, which provided sufficient degree of abrasion to damage the endothelium but not the vascular SMC layer. After reviewing the aforementioned methods, we decided to use human hair to mechanically abrade endothelium from preglomerular and mesenteric arteries in order to investigate the role of endothelium in MC.

### Role of Endothelium in MC

Even though MC is essential in renal autoregulation, its dependency on endothelium is controversial. Studies in different species and tissues have reported that endothelium removal abolished (Harder, 1987; Katusic et al., 1987; Rubanyi, 1988; Kuo et al., 1990; Hughes and Bund, 2002), enhanced (Garcia-Roldan and Bevan, 1990; Juncos et al., 1995; Scotland et al., 2001; Gschwend et al., 2003; Huang et al., 2005; Chaston et al., 2013), reduced (Huang et al., 2005), or did not change (Hwa and Bevan, 1986; Falcone et al., 1991; Scotland et al., 2001; Daneshtalab and Smeda, 2010) MC. This controversy may be because endothelial cells can release different vasoactive substances in variable quantities in different vascular beds or even different sections of the same vascular bed (Ferrer et al., 1995; Thorin et al., 1997; Hill et al., 2001). Thus, it is important to investigate the function of endothelium in different vascular beds. In rabbit afferent arterioles, (Juncos et al., 1995) reported that removing endothelium enhanced MC in free-flow vessels but did not change MC in no-flow arteries. Nevertheless, the authors used factor VIII-related antigen antibodies which did not completely remove the endothelium, revealed in their transmission electron micrographs (Juncos et al., 1995). Another study conducted by Harder demonstrated a dependency of MC on endothelium in cat cerebral arteries. Harder subjected these arteries to 40 to 60 mmHg pressure and recorded a depolarization of 0.35 mV/mmHg, which was abolished when he removed the endothelium (Harder, 1987). In mesenteric arteries, endothelial cells may have roles in MC under hypoxic conditions. Earley and Walker (2002) exposed Sprague-Dawley rats to chronic hypoxia and found that myogenic responsiveness in their mesenteric arteries was abolished. This obliteration was restored by removing the endothelium.

### NSAIDs in Hypertension and CKD

Non-steroidal anti-inflammatory drugs (NSAIDs) are a class of drugs that block prostaglandins synthesis by inhibiting COX-1 and COX-2 enzymes. While healthy people rarely have adverse renal side effects upon using NSAIDs, individuals (particularly elderly patients) who have hypertension and CKD may develop acute kidney failure (Hörl, 2010). In fact, administering indomethacin to renal failure rat models (Sprague–Dawley rats that have been administered adenine to have chronic renal failure) significantly decreased their survival rate (Kadowaki et al., 2012). Every year, about 2.5 million Americans who use NSAIDs experience renal-related side effects (Sandhu and Heyneman, 2004).

## Conclusion

Myogenic constriction is augmented in the SHR preglomerular arteries but not the mesenteric arteries, compared to the WKY respective vessels. The augmented MC in the SHR pre-glomerular vessels appears to be due to increased prostanoid production, particularly TXA2 synthesis, from the tunica media and/or adventitia layers. Moreover, MC is not dependent on the endothelium in the WKY and SHR pre-glomerular and mesenteric arteries, as removing endothelium did not change the MC. L-type Ca^2+^ channels are critical to MC as inhibiting them tremendously decreases MC in both WKY and SHR arcuate and mesenteric arteries. Nevertheless, SHR pre-glomerular arteries seem to have a differential intracellular Ca^2+^ signaling at higher intraluminal pressures (P140-P180 mmHg) compared to the WKY vessels.

## Data Availability Statement

All datasets generated for this study are included in the article/supplementary material.

## Ethics Statement

The animal study was reviewed and approved by McMaster University Animal Research Ethics Board.

## Author Contributions

SN and CL performed the experiments, analyzed the data, and interpreted the results. SN prepared the figures and drafted the manuscript. SN and JD edited and revised the manuscript. All authors approved the final version of the manuscript, contributed to the article and approved the submitted version. JD conceived and designed the research.

## Conflict of Interest

The authors declare that the research was conducted in the absence of any commercial or financial relationships that could be construed as a potential conflict of interest.
